# Parametric Design and Finite Element-Based Structural Assessment of Industrial Moulds for Concrete Blocks

**DOI:** 10.3390/ma19163494

**Published:** 2026-08-18

**Authors:** Erick Tatayo-Tipantasi, Víctor Erazo-Arteaga, Paul Tafur-Escanta, Juan P. Tafur, Robert Valencia-Chapi

**Affiliations:** 1 Facultad de Ingeniería en Ciencias Aplicadas, Universidad Técnica del Norte, Ibarra 100150, Ecuador; eatatayot@utn.edu.ec (E.T.-T.); vaerazo@utn.edu.ec (V.E.-A.); 2 Facultad de Ingeniería en Ciencias Agropecuarias y Ambientales, Universidad Técnica del Norte, Ibarra 100150, Ecuador; pmtafur@utn.edu.ec; 3 Departamento de Mecánica, Química y Diseño Industrial, ETSIDI, Universidad Politécnica de Madrid, 28012 Madrid, Spain; jp.tafur@upm.es

**Keywords:** CAD-CAE design, fatigue safety factor, finite element method, industrial moulds, injection point, INEN-3066 standard, parametric modelling

## Abstract

**Highlights:**

- A standardised parametric-modelling workflow, driven by QFD/VOC matrices, links customer and regulatory requirements directly to CAD control variables for concrete-block moulds.
- Finite element analysis identifies the 10 cm female die as the critical structural component, whose fatigue safety factor falls below the required threshold of 1.0 at just 4000 N, well below the maximum evaluated overload (10,000 N).
- Male components maintain fatigue safety factors above 1.61 even under the maximum evaluated overload (10,000 N), confirming that failures originate from overload rather than routine use.
- A balanced 19 mm gating system fills the mould cavity in under 0.11 s, demonstrating the feasibility of an ultrafast pressurised injection cycle.

**Abstract:**

The conventional fabrication of concrete block moulds is characterised by persistent challenges related to standardisation, protracted redesign processes, and an absence of structural validation, all of which undermine regulatory compliance. This study proposes a standardised parametric modelling process aimed at ensuring compliance with the technical criteria of the INEN-3066 and ASTM C90 standards. An integrative methodology combining QFD/VOC matrices with CAD-CAE tools was used to parameterise three commercial mould configurations (10, 15, and 20 cm) in SolidWorks 2023. A finite element analysis (FEA) was subsequently conducted in ANSYS 2025 R1 under iterative overloads of up to 10,000 N, complemented by a rheological analysis in SolidWorks Plastics. The results show that the “male” (punch) components exhibit consistently high stiffness, maintaining fatigue safety factors above 1.61 across all three configurations. In contrast, the “female” (die) components are the more vulnerable link in the assembly: the fatigue safety factor of the 10 cm die drops below the required threshold of 1.0 at 4000 N, compared with 6175.6 N and 9254 N for the 15 and 20 cm configurations, respectively. The rheological analysis further confirmed the feasibility of an ultrafast injection cycle, with cavity filling times below 0.11 s and injection pressures ranging from 6.105 to 12.9 MPa across all formats. It is posited that, in accordance with the parametric model, a reinforced-wall geometry should be adopted for the 10 cm die, characterised by an augmentation of wall thickness by 15% and enlarged fillet radii. This is projected to elevate its fatigue-critical load beyond 4500 N without necessitating any alteration in the external block dimensions. These findings indicate that parametric CAD-CAE-CFD digitalisation can anticipate structural failures before manufacturing, offering a computational pathway toward regulatory compliance that should be confirmed through physical prototype testing.

## 1. Introduction

In Ecuador, the construction sector contributes 7% of the gross domestic product, with 4048 buildings projected for 2026. In these projects, concrete blocks account for 46% of the masonry used. However, 95% of block manufacturers operate informally or on an artisanal basis, while fewer than 5% hold official certifications [1,2,3]. Artisanal manufacturing limits the control of raw materials, mix proportions, compaction, and the dimensions of the finished product. As a result, some of the blocks marketed in the country do not meet the minimum compressive strength values established in technical standards [4]. This situation affects the quality of the elements used in masonry and highlights the need to control their manufacturing conditions.

ASTM C90 establishes the requirements for the manufacture, testing, classification, and acceptance of structural concrete blocks. Its provisions include dimensional tolerances, minimum wall and web thicknesses, and the compressive strength values required for acceptance [5]. At the national level, NTE INEN 3066 defines the quality requirements and test methods applicable to concrete blocks. It also classifies these elements according to their use and establishes the modular and nominal dimensions that must be considered during their manufacture and use in construction works [6]. Compliance with these provisions depends on the conditions applied during the production process. Mix proportioning, the introduction of fresh concrete, compaction, and mould geometry influence the dimensions and mechanical strength of the block. Mould precision affects cavity uniformity, element thickness, and the formation of the material’s internal structure [7].

Mould design can be optimized using design and simulation tools. Currently, CAD and CAE engineering tools make it possible to model, simulate, and modify components before manufacturing. The integration of SolidWorks 2023 and ANSYS 2025 R1 enables the development of parametric models and the evaluation of different geometric configurations. The adoption of this approach facilitates structural optimization and reduces the need to manufacture multiple physical prototypes [8]. The application of these tools in the construction sector has made it possible to compare structural configurations and evaluate alternatives before implementation. Li et al. [9] studied the strengthening of prestressed concrete box-girder bridges using finite element analysis and load tests. The results showed a 24.55% reduction in maximum deflection and an 11.57% improvement in transverse load distribution using concrete-filled steel tube trusses. Tyflopoulos and Steinert [10] combined parametric optimization with finite element analysis in a perforated plate, an L-bracket, and an MBB beam. The authors achieved mass reductions ranging from 96.9% to 99.1% without exceeding the established stress limit. The study made it possible to compare different geometric configurations and select lower-mass alternatives before manufacturing.

Teschemacher and Bletzinger [11] integrated parametric CAD design with the structural analysis of modular constructions. The models were divided into several configurations to evaluate the influence of the number and arrangement of the connections. In one of the beams analysed, deflection increased from 0.833 mm in the continuous model to 2.124 mm and 5.126 mm in the modularized models. These results show that geometric changes and connections can be evaluated directly during the design process. In the case of concrete blocks, Sambucci et al. [12] compared conventional elements and blocks manufactured with recycled rubber through simulations and compression tests. The numerical model reproduced the trend of the experimental behaviour, although it underestimated the strength, with approximate agreement levels of 45% and 55%. The authors related these differences to drying shrinkage, cavity accuracy, variations between the actual and modelled geometries, and the representation of concrete as a homogeneous material.

The structural analysis of the mould must be complemented by the study of fresh concrete behaviour during filling. Computational fluid dynamics makes it possible to represent material displacement, its interaction with the mould walls, and the geometry obtained after the deposition process. El Abbaoui et al. [13] analysed mortar extrusion and deposition using CFD, considering different rheological models and slip conditions. The results indicated that an inadequate definition of these conditions alters the predictions of flowability, deformation, and the final dimensions of the element. The study indicates that geometric representation must be accompanied by an appropriate description of the material rheology and its interaction with the contact surfaces. Comminal et al. [14] used CFD models to analyse the flow of fresh concrete and estimate the resulting geometry. The mortar was represented using the Bingham model, in which the material begins to flow when the applied stress exceeds the yield stress. Beyond this point, its behaviour depends on the plastic viscosity. Dimensional deviations were associated with surface irregularities, fluctuations in the inlet flow rate, and differences between the idealized model and the actual behaviour of the mortar.

The reviewed literature applies finite element analysis to the optimization of structures and components in the construction sector. It also uses CFD models to study the rheological behaviour of fresh concrete during extrusion and deposition processes. However, these approaches have been developed separately and focus on finished elements, structural systems, or generic geometries. No parametric methodologies were identified that integrate the design of industrial moulds for concrete blocks, particularly with respect to specific compliance with NTE INEN 3066. This absence limits the joint evaluation of mould geometry, mechanical strength, and material behaviour during filling. To contextualize the present study and delineate the research gap, Table 1 summarizes the analysed literature, highlighting its findings, limitations, and the identified research gap.

The objective of this study is to develop and evaluate a parametric model for concrete block moulds in accordance with the specifications of NTE INEN 3066 and industrial manufacturing criteria. The structural and fluid-dynamic behaviour of three commercial configurations of 10, 15, and 20 cm was evaluated under the compaction loads applied by block-making machines using an integrated CAD-CAE-CFD workflow. The results made it possible to establish the fatigue safety limits of the structural dies, with a value of 4000 N for the 10 cm mould, and to determine material inlet parameters aimed at reducing production cycle times.

## 2. Materials and Methods

The methodology followed a mixed-methods approach, integrating documentary analysis with an experimental design based on digital simulation. The study was structured into five phases to optimize the parametric variables and assessed the structural integrity of metal moulds used for manufacturing concrete blocks. Three standard commercial mould configurations, with widths of 10, 15, and 20 cm, were evaluated throughout the study.

### 2.1. Documentary Review and Functional Requirements

The mould design was based on a comparative analysis of the Ecuadorian standard NTE INEN-3066 [6] and the U.S. standard ASTM C90 [5]. INEN-3066 establishes strict dimensional requirements for hollow concrete blocks in Ecuador, including a maximum tolerance of ±3 mm for nominal dimensions (width, height, and length) and a minimum compressive strength. ASTM C90 complements these criteria by specifying requirements for load-bearing masonry units, particularly the minimum wall thickness (webs and shells) needed to prevent failure by buckling or crushing. Together, these standards require the metal mould to remain essentially non-deformable under service loads to ensure that the manufactured blocks comply with the specified dimensional tolerances.

To translate regulatory requirements and operational needs into engineering parameters, a sequence of quality-oriented methods was applied. The Voice of the Customer (VOC) was first captured through interviews with technical staff, identifying recurring problems such as premature wear and mould deformation. This qualitative information was filtered through a Critical-to-Quality (CTQ) diagram, which defined three pillars: dimensional stability, durability, and equipment weight. These pillars were then incorporated into a Quality Function Deployment (QFD, or “House of Quality”) matrix, following the foundational methodology originally proposed for translating customer requirements into engineering characteristics [15]. The statistical analysis of the QFD correlated customer requirements with technical variables, identifying the structural thickness of the mould partitions and walls, together with the creep resistance of the material, as the priority factors for design control, because of their higher relative weighting.

### 2.2. Parametric CAD Modelling

The three-dimensional design of the dies (“female”) and punches (“male”) was carried out in SolidWorks (Dassault Systèmes). To provide design versatility, a parametric model was developed and managed through dimensions, geometric relations, and design tables. This structure makes it possible to generate different configurations and update dependent operations when the parameters defined in the model are modified, without compromising existing constraints, and enables scalability across the 10, 15, and 20 cm mould sizes [16]. The critical geometric variables entered into the system are summarised in Table 2.

### 2.3. Structural Simulation (FEA)

Mechanical integrity was assessed using finite element analysis (FEA) in ANSYS, following standard finite element formulation and discretisation principles [17,18]. To define realistic boundary conditions, the compaction forces exerted by the block-making machine on the mould were first calculated analytically using the law of levers ($F_{1}\cdot (d_{1}+d_{2})=F_{2}\cdot d_{2}$) and the moment equilibrium of the machine’s mechanical system. Operating loads applied to the flat-top surface ranged from 1960 N to 2254 N, depending on the mass of the mould (see Table 3). The spatial domain was discretised using a high-quality, fine mesh averaging 96,000 elements, and the model was subjected to iterative overloads applied in increments up to 10,000 N. The output variables analysed for regulatory compliance were the equivalent von Mises stress (MPa), the maximum deformation (mm), and the structural fatigue safety factor (required to be ≥1).

**Figure 1:**
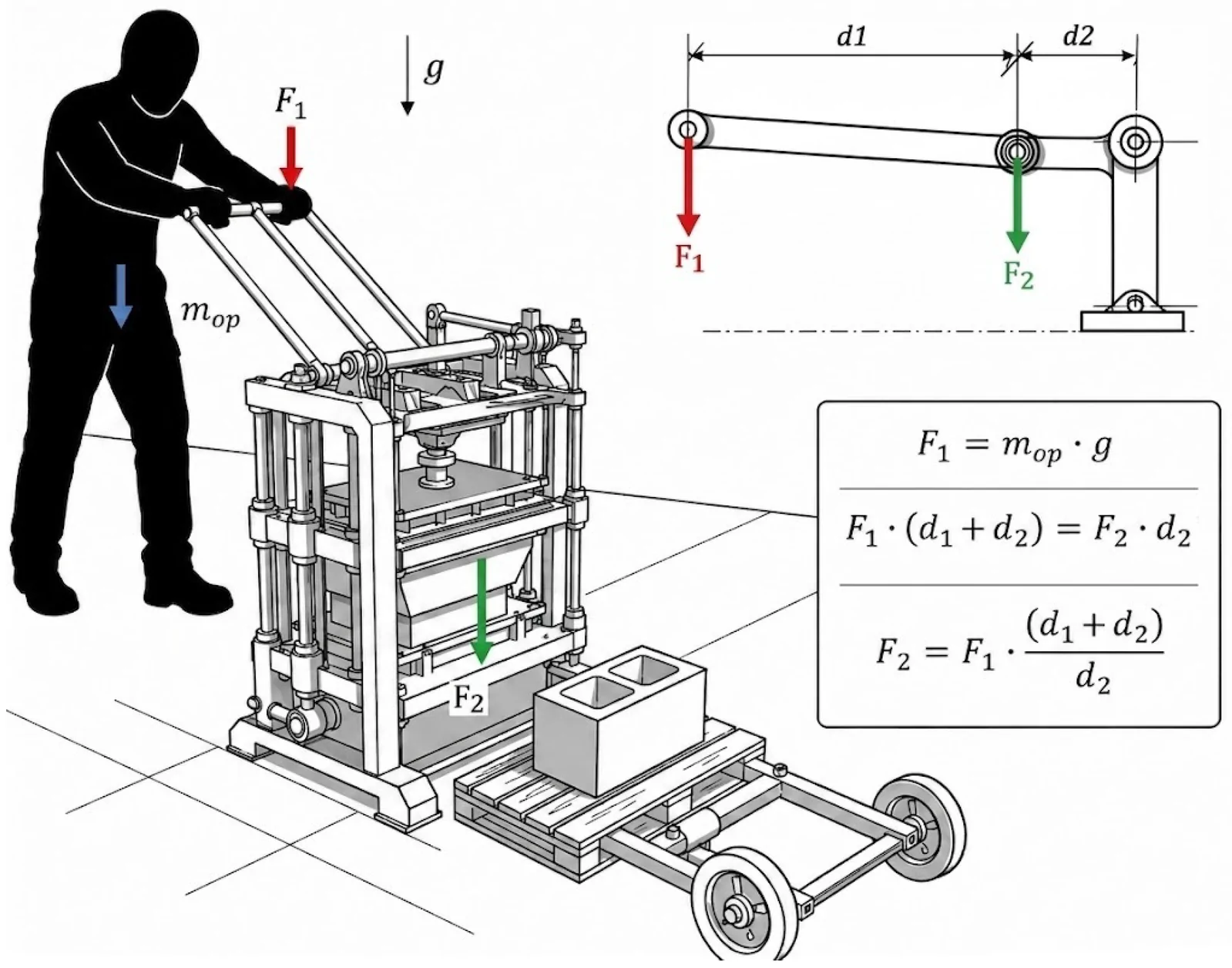
Mechanical and analytical models of the manual concrete block-making machine. The schematic illustrates how the operator’s gravitational force ($F_{1}=m_{op}\cdot g$) is mechanically amplified through the lever arms $d_{1}$ and $d_{2}$ when the operator transfers their full body weight to the lever, generating the axial compaction force ($F_{2}$) that is transmitted to the mould assembly.

In an effort to accurately establish the mechanical boundary conditions of the finite element model, the axial compaction force transmitted to the mould assembly ($F_{2}$) was determined through an analysis of the machine’s lever mechanism (see Figure 1). The primary driving force ($F_{1}$) is derived from the operator’s body mass ($m_{op}$), which is subject to gravitational acceleration (g). In accordance with the principle of static equilibrium of moments (∑M = 0) with regard to the fixed support, the relationship between the applied input force ($F_{1}$) and the resulting compaction force ($F_{2}$) is governed by the law of levers $F_{1}\cdot (d_{1}+d_{2})=F_{2}\cdot d_{2}$, where $d_{1}$ and $d_{2}$ denote the lever arms of the driving force and the resistance, respectively. It is assumed that the operator’s mass, $m_{op}$, is 75 kg. This is consistent with the manufacturer’s operating specifications for the manual block-making machine used at the host facility. The application of these values in $F_{2}=F_{1}\cdot \frac{\left(d_{1}+d_{2}\right)}{d_{2}}$ yielded the operating loads of 1960.0 N, 2100.5 N, and 2254.0 N reported in Table 3 for the 10, 15, and 20 cm moulds, respectively. The minor discrepancies observed between the formats arise because the self-weight of each mould configuration is added to the lever-amplified force to yield the total operating load $F_{2}$. The analysis was conducted through the implementation of iterative overloads, with a maximum of 10,000 N applied, thereby ensuring a safety margin that exceeds fourfold the nominal operating force. No formal dynamic amplification factor was derived from the machine’s vibration spectrum or compaction kinematics; instead, the overload sweep was used as a conservative bounding envelope for the combined effect of compaction dynamics and vibration-induced peaks. The characterisation of the actual dynamic/vibratory load spectrum of the compaction process, for instance using accelerometry on the physical machine, is identified as a priority for future experimental work (Section 4.2).

Two categories of load were defined for this study. Operational loads correspond to the actual, nominal compaction forces applied by the machine under normal operating conditions and vary according to the mould’s mass and shape. Iterative (overload) loads, by contrast, were applied virtually in increments of up to 10,000 N, both to determine the fatigue-critical load at which each die’s safety factor fell below the required threshold, and, at the most extreme step evaluated, to drive the material to its actual yield point. The loads applied to the flat upper surface of the ram are detailed in Table 3.

The dies and punches were modelled as isotropic linear-elastic structural steel, using the default material properties provided in the ANSYS Engineering Data library (Table 4). These values were adopted directly from the software library rather than from mill-certified test data for the specific tool steel used in production; this is noted here as a limitation of the present analysis, and the use of certified material data is recommended for subsequent, higher-fidelity iterations of this model.

**Figure 2:**
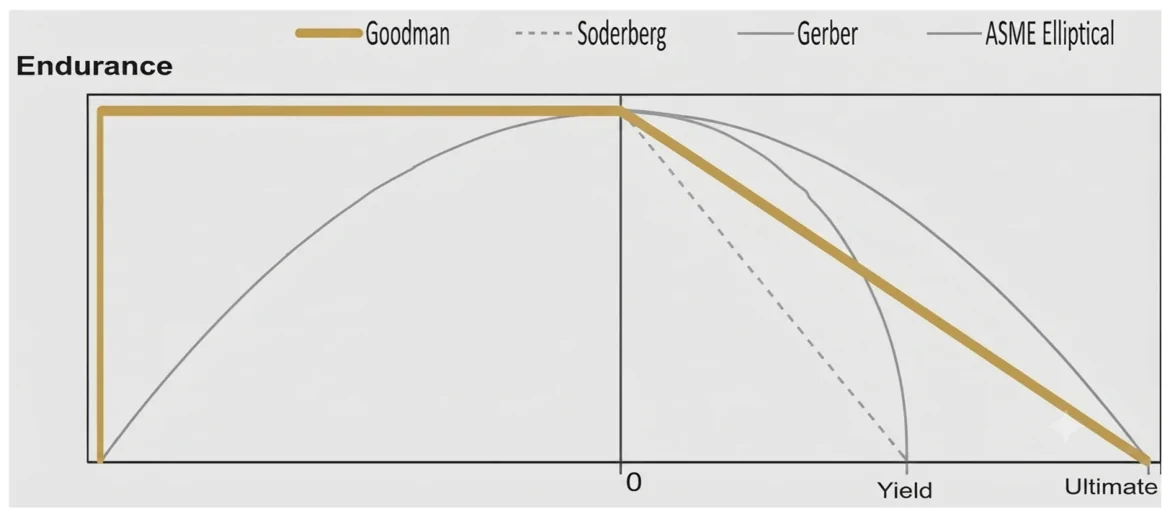
Theories of average stress correction have been evaluated in ANSYS. The Goodman curve (highlighted in yellow) was selected to govern the fatigue analysis and estimate the safety factor for a design life of 5 × 10^6^ load cycles with a zero point.

To accurately assess the long-term structural viability of the moulds under continuous industrial operating conditions, a life cycle analysis was performed using the ANSYS Fatigue Tool. ANSYS Workbench provides tools for managing engineering data and the properties of the materials used in simulation models, facilitating their incorporation into the analysis workflow [18]. The cyclic compaction process was modelled using a “Zero-Based” load type, simulating the repeated compression and release action of the block-making machine. The target design life was set at 5 × 10^6^ cycles. This target was selected as a conservative fatigue design criterion: under the company’s single-shift operating schedule (8 h/day, 5 days/week) and a compaction cycle time of 10–15 s, 5 × 10^6^ cycles corresponds to approximately 6.7–10.0 years of continuous production, exceeding the facility’s expected mould replacement interval of under 5 years and thereby providing an additional margin against fatigue failure. Among the available mean-stress correction criteria (Figure 2), the Goodman line was selected as it provides a linear, conservative estimate widely adopted in mechanical design practice for ductile structural steels [19]. This ensures that the calculated safety factors reflect the actual operating limits of the equipment during prolonged use.

### 2.4. Mesh Quality

**Figure 3:**
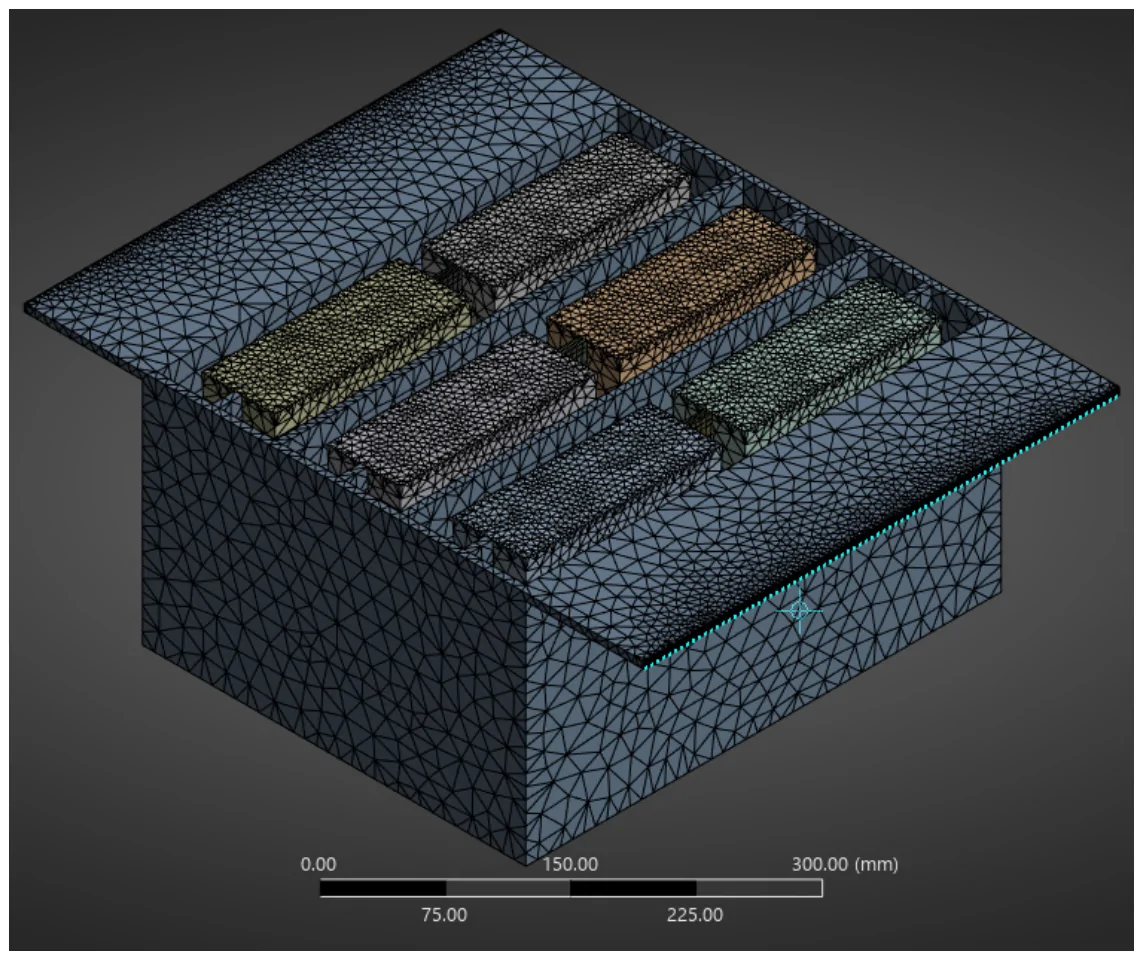
The discretization of the 10 cm female die spatial domain was achieved through the utilization of high-order tetrahedral solid elements in ANSYS. The model incorporates local mesh refinement at internal edges and transition zones to accurately capture stress concentrations.

To guarantee the precision and consistency of the FEA results, a mesh quality-control procedure was carried out within ANSYS. The spatial domain of the moulds was discretised using high-order tetrahedral solid elements, well suited to capturing the complex geometries and fillet radii of the dies. Local refinement (mesh control) was applied to internal edges and transition zones where higher stress concentrations were expected, producing a final mesh averaging 96,000 elements. Two standard quality metrics were used to validate the mesh: skewness, which remained below 0.85 (considered excellent), and orthogonal quality, which recorded values above 0.75, together ensuring that the stress and strain results were free of numerical singularities or artefacts (see Figure 3). A dedicated h-refinement convergence study was not performed at the internal-corner locations where peak stresses were recorded, a limitation that is acknowledged in Section 4.2.

### 2.5. Model Constraints and Boundary Conditions

**Figure 4:**
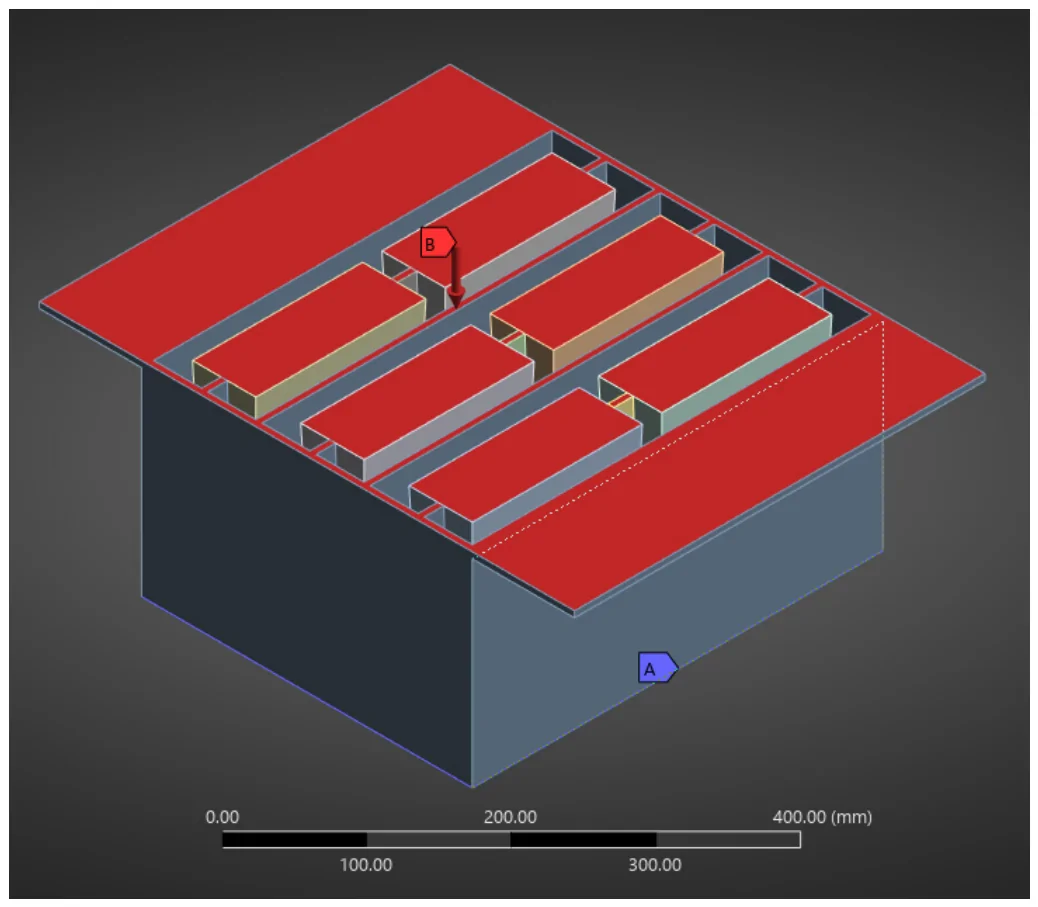
Static structural boundary conditions and model constraints have been implemented in ANSYS. The application of fixed support to the lower base plate (Ux=Uy=Uz=0) is intended to prevent displacement of the rigid body. The downward axial compaction force is applied uniformly to the upper surfaces of the mould assembly. Label A is the fixed support, and Label B is the applied compaction force.

In order to simulate the actual operating environment of the manual concrete block-making machine, explicit structural boundary conditions and displacement constraints were applied in the ANSYS Static Structural environment (see Figure 4). A fixed support (label A, highlighted in blue) was assigned to the entire bottom surface of the mould’s base plate, thereby restricting all degrees of freedom of translation and rotation (Ux=Uy=Uz=0) in order to represent the machine’s rigid support base and to prevent unrestricted movement of a rigid body during the structural analysis.

A downward axial compaction load (label B, highlighted in red) was uniformly distributed along the upper horizontal surfaces of the mould assembly, simulating the maximum consolidation load applied during the block forming phase. The magnitude of this load, when considered in isolation, accounts for the amplified mechanical advantage of the lever mechanism, as well as dynamic overloads.

Furthermore, specific structural simplifications and interfacial behaviours were defined with the objective of ensuring computational convergence and physical accuracy. Welded and bolted fasteners: The structural joints connecting the lower base plate, the internal partition walls, and the outer walls of the mould, which physically correspond to welded joints, were represented as bonded contacts. This provided equivalent interfacial stiffness without introducing artificial stress singularities at the locations of the fasteners. In the structural analysis, the concrete mixture and the punch were not modelled as discrete contact bodies; rather, their combined mechanical effect during compaction was represented by the equivalent distributed pressure (label B) applied directly to the mould’s upper surfaces. This pressure was derived from the lever-mechanism calculation described in Section 2.3. This simplification is discussed further as a modelling limitation in Section 4.2.

### 2.6. Computational Fluid Dynamics (CFD)

The rheological analysis was performed using the SolidWorks Plastics module to evaluate the mould-filling behaviour and the feasibility of manufacturing the part by injection moulding [20]. The material’s dynamic viscosity and slump parameters were calibrated using the consistency and slump criteria established in ASTM C143 (Standard Test Method for Slump of Hydraulic-Cement Concrete) [21], enabling an accurate simulation of the pressures and filling times within the mould cavity. Fresh concrete was treated as a Bingham plastic material, consistent with established rheological descriptions commonly applied to concrete flow and slump-test analysis [22,23]. To ensure multiphysical consistency between the structural and rheological analyses, the thermomechanical properties of concrete used to configure the material domain in SolidWorks Plastics were sourced from the ANSYS Engineering Data library’s standard concrete material entry (rather than from SolidWorks Plastics’ own default database), so that both analyses shared a common material reference; these parameters are critical for calculating mixture distribution and energy transfer during injection [20,24].

The pour system was configured with a balanced gating layout and a central injection point 19 mm in diameter, branching symmetrically towards the mould cavities. The fluid domain was discretised with a high-density volumetric mesh to accurately track the progression of the flow front, and boundary conditions included thermal insulation of the steel walls and a regulated injection temperature for the mixture. The software’s thermal input was set as a proxy for the fresh-concrete process temperature, rather than representing an actual melting point. The simulation evaluated the viscoelastic behaviour of the concrete to determine the maximum filling time and the injection pressure required for uniform distribution within the cavities without air-pocket formation. The required injection pressure was found to scale with mould volume, ranging from 6.105 MPa for the 10 cm mould to 12.9 MPa for the 20 cm mould. The filling-front algorithm was additionally used to predict and mitigate typical manufacturing defects, ensuring uniform volumetric consolidation, preventing critical weld lines, and confirming the complete evacuation of air traps that could otherwise compromise the block’s compressive strength.

### 2.7. Ergonomics and Operational Efficiency of the Injection System

The proposed computerised injection system also has direct implications for ergonomics and industrial productivity. Replacing traditional manual vibration compaction with a pressure-controlled fluid injection system reduces operator exposure to continuous mechanical vibration and heavy-load handling, lowering the risk of fatigue and musculoskeletal disorders. At the same time, the model’s thermal and rheological efficiency ensures that cavities fill in under 0.11 s, a substantial acceleration that shortens manufacturing cycle times per block, increasing production volume and improving the overall profitability of the metalworking process.

## 3. Results

The computational analysis of the parametric model demonstrated its mechanical and fluid dynamic feasibility. All three mould configurations satisfied the requirements specified in INEN-3066 and ASTM C90 standards.

### 3.1. Structural Analysis (FEA) of the Mould Components

The finite element analysis results show significant differences in mechanical stiffness between the compaction equipment components.

#### 3.1.1. Strength of the Male Component (Punch)

**Figure 5:**
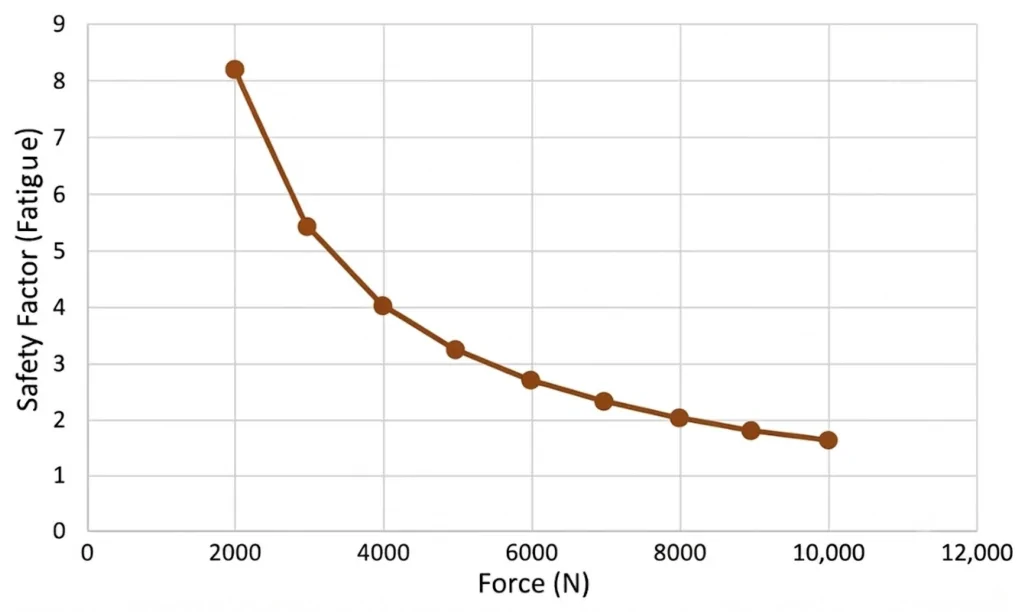
Force–fatigue-safety factor relationship for the 10 cm male mould, shown as the representative (most critical) case among the three punch configurations evaluated.

The simulations demonstrated a highly robust structure across all three punch configurations. Under the maximum evaluated overload (10,000 N), the 15 and 20 cm punches maintained fatigue safety factors above the required minimum, reaching 2.05 and 2.71, respectively. The 10 cm punch, by contrast, exhibited a fatigue safety factor of 1.61 at 10,000 N, marginally above the required minimum of 1. Figure 5 illustrates this force–fatigue-safety factor relationship for the 10 cm punch, selected as the representative case since it is the most structurally demanding of the three configurations; the 15 and 20 cm punches follow the same monotonic trend at correspondingly higher safety-factor levels (2.05 and 2.71 at 10,000 N, respectively). It was observed that the material remained within stable and safe limits when subjected to overloads of up to 10,000 N. This point is discussed in greater detail in Section 4.

#### 3.1.2. Vulnerability of the Female Component (Die)

In contrast, the dies, or female components, represent the critical link in the design. The 10 cm configuration was the most vulnerable, falling below the required fatigue safety threshold of 1.0, reaching a minimum of 0.91, once the compaction load reached 4000 N. For the 15 and 20 cm moulds, the fatigue safety threshold was exceeded at 6175.6 N and 9254 N, respectively. Even so, the maximum deformation recorded across all safe operating scenarios remained strictly below 0.2 mm, confirming that the design meets the dimensional-tolerance requirements of the INEN-3066 standard within its normal operating range (Table 5). It is important to note that the safety factor values reported in Table 5 do not precisely equal 1.0 at the critical load. This is because they correspond to the closest simulated load increment at which the threshold is crossed, rather than an exact interpolated intercept. The slightly lower limit value recorded for the 15 cm die (0.88) compared with the 10 and 20 cm configurations (0.91 and 0.92, respectively) reflects small geometry-dependent differences in local stress concentration among the three formats, rather than a non-monotonic trend in structural performance; the 15 cm die still reaches this threshold at a substantially higher load (6175.6 N) than the 10 cm die (4000 N).

**Figure 6:**
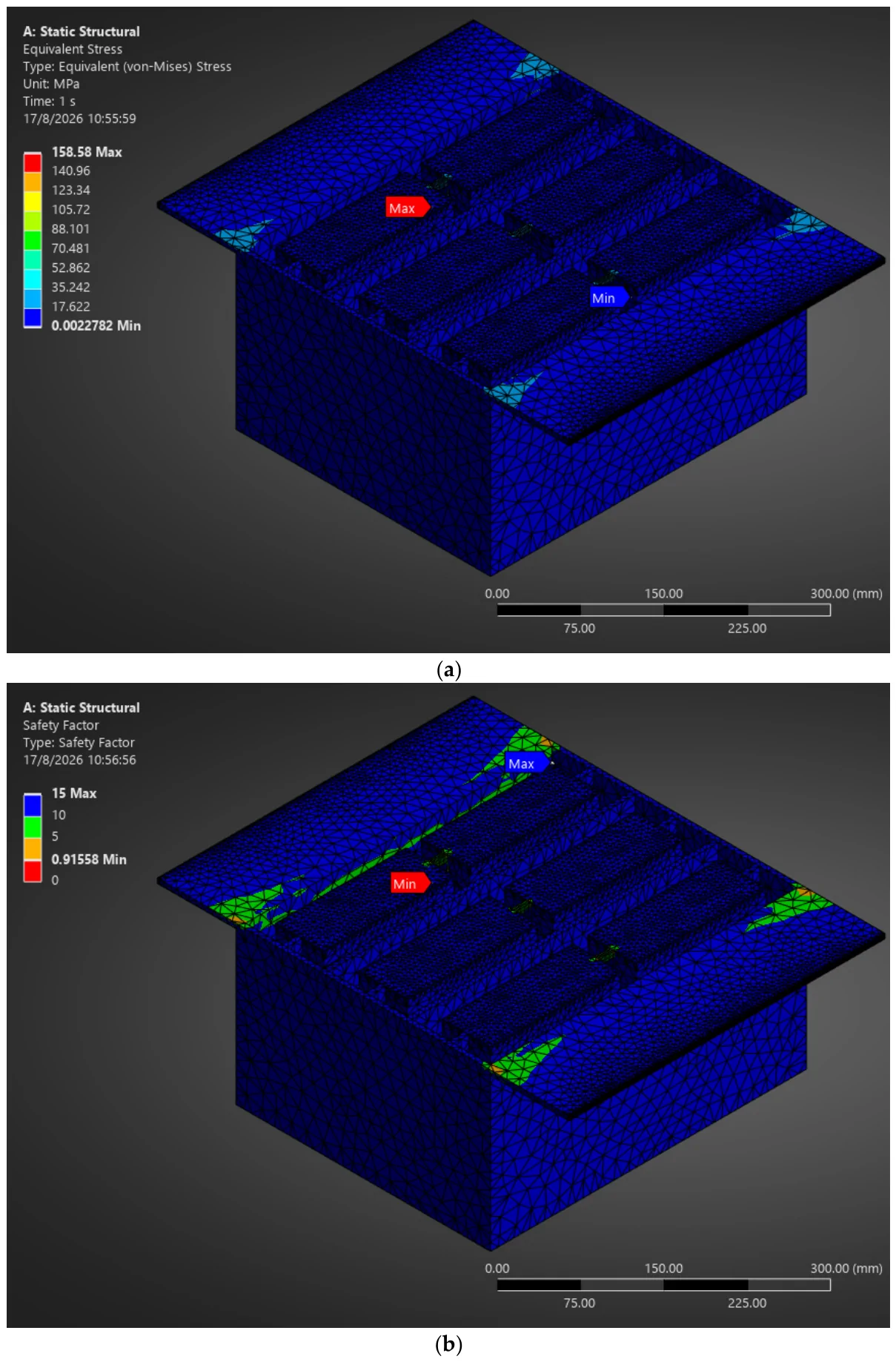
Analysis with ANSYS. (**a**) Equivalent von Mises stress distribution at the failure point of the 10 cm female die under a load of 4000 N, reaching a critical peak of 158.58 MPa. (**b**) Fatigue-safety factor distribution at the same load, showing the regions (in red) where the value dropped to 0.91, the point at which the fatigue safety threshold is no longer met.

Figure 6 presents the structural simulation results for the 10 cm female die at its failure point, under a compaction load of 4000 N. Figure 6a shows the equivalent von Mises stress distribution, with a maximum value of 158.58 MPa concentrated at the upper corners of the internal partition walls, while the lowest stress levels appear across the base of the component. Figure 6b displays the corresponding fatigue safety factor distribution for the same loading condition: the minimum value drops to 0.91 along the outer side walls, falling below the required safety threshold of 1 and confirming that the fatigue safety factor drops below the required limit in that region, whereas the innermost, less-loaded areas retain fatigue safety factors as high as 15.

#### 3.1.3. Comparative Mechanical Behaviour

**Figure 7:**
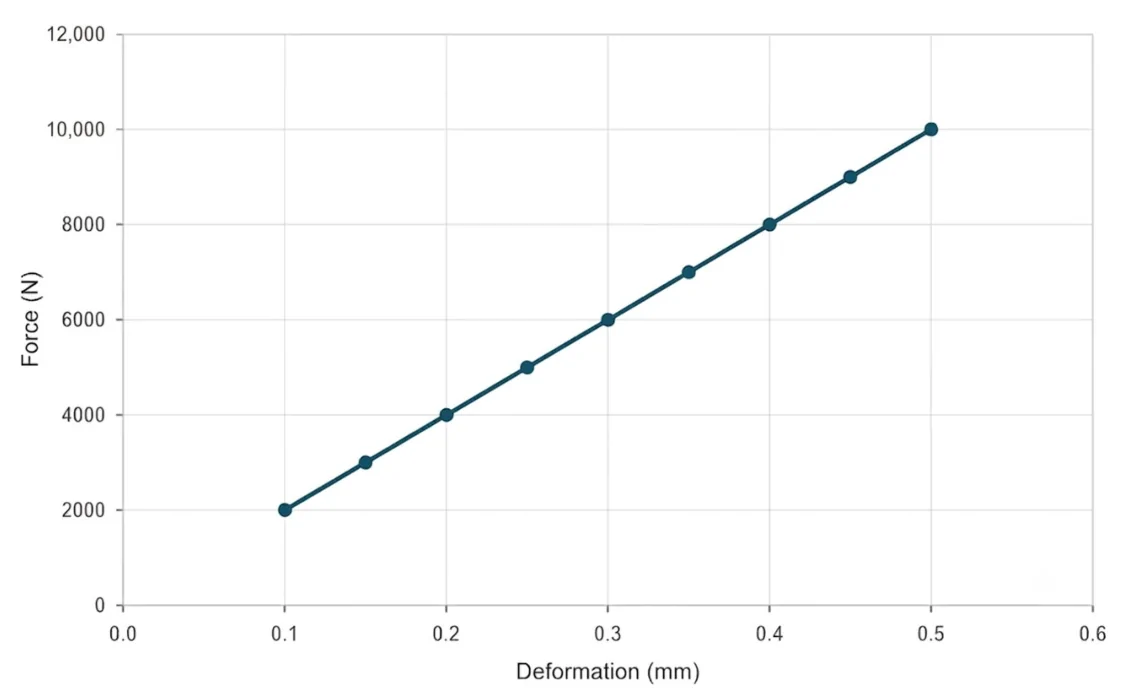
Force–deformation curve for the 10 cm female component, showing a steep slope that approaches the 0.2 mm dimensional-tolerance limit at relatively low loads.

**Figure 8:**
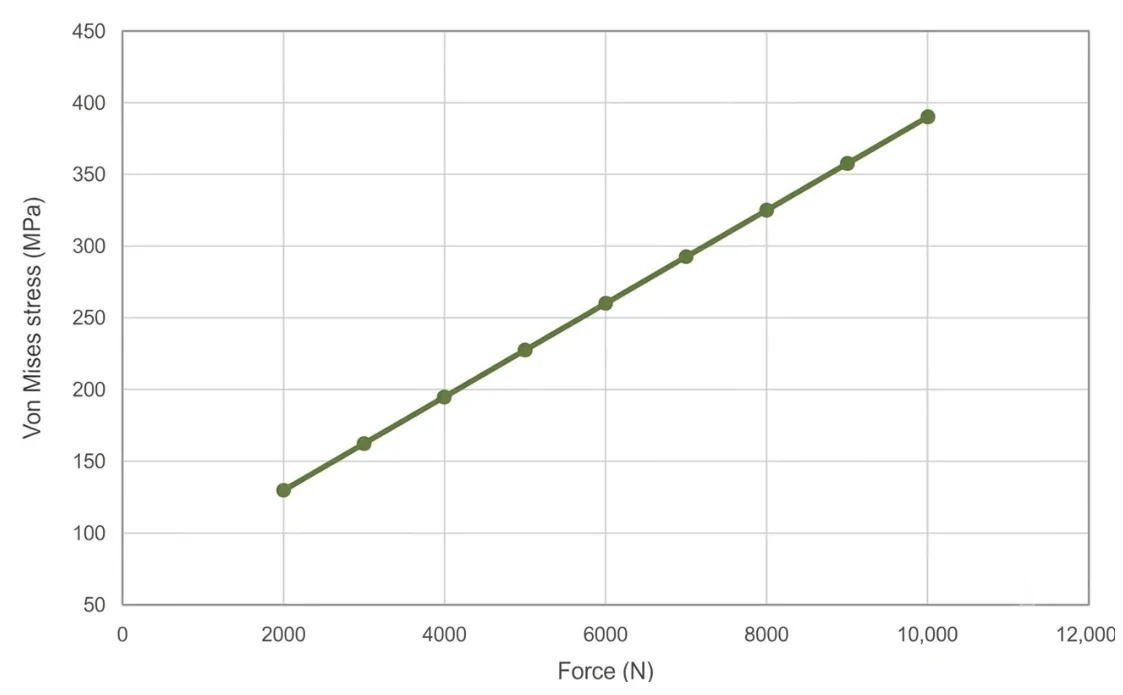
Force–stress relationship for the 10 cm female mould. Von Mises stress rises sharply with applied load, exceeding the 250 MPa yield strength of the steel beyond approximately 6500 N; results beyond this point fall outside the valid range of the linear-elastic model and are shown for trend purposes only.

**Figure 9:**
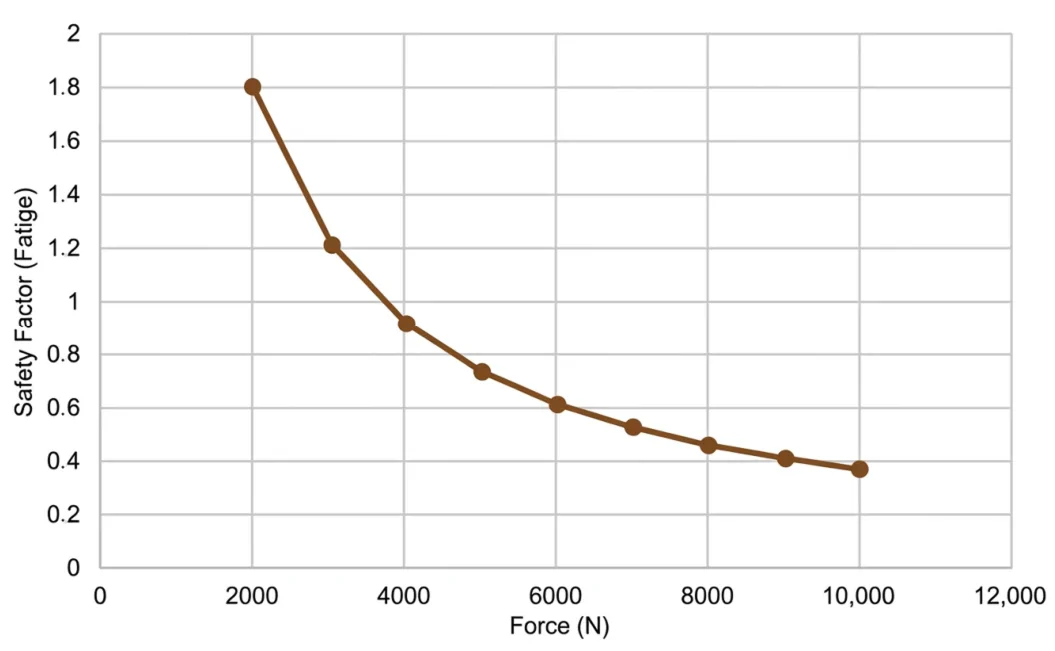
Force–fatigue-safety factor relationship for the 10 cm female mould. The curve falls below the safe threshold of 1.0 when it reaches 4000 N, confirming the die’s vulnerability under overload conditions.

To better characterise the dynamic behaviour of the parametric design, the progression of deformation (Figure 7), von Mises stress (Figure 8), and fatigue safety factor (Figure 9) were evaluated as the applied force increased from the operating load to the maximum evaluated overload (10,000 N).

Comparing force against von Mises stress shows that the 10 cm configuration rapidly concentrates stress in its transverse webs, exceeding the yield strength of structural steel considerably sooner than the 15 and 20 cm configurations (Figure 8).

Finally, the force-to-fatigue-safety factor ratio confirms that, irrespective of commercial format, the punches maintain a fatigue safety factor above 1.61 throughout the evaluated load range, while the dies fall into critical ranges (below 1.0) once they exceed their respective fatigue safety limits (Figure 9).

### 3.2. Computational Fluid Dynamics and Injection Simulation (CFD)

**Figure 10:**
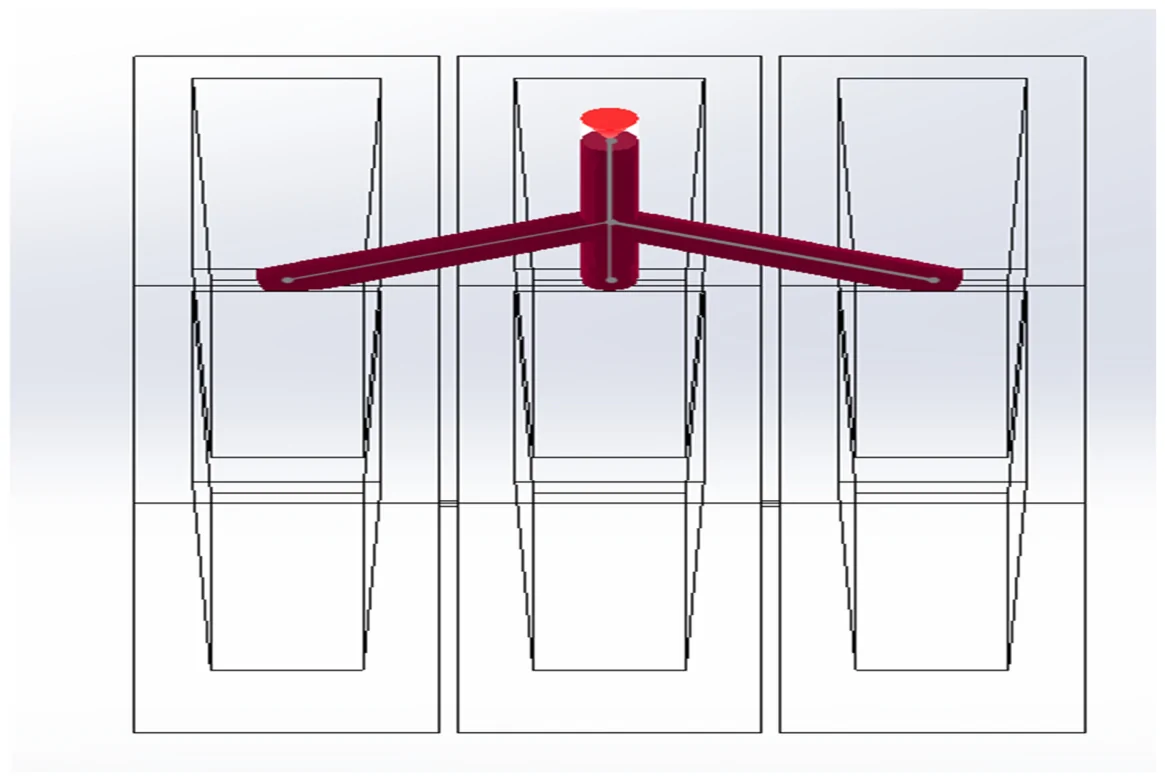
Geometric configuration of the balanced gate design is characterized by a central injection point measuring 19 millimetres, which branches symmetrically toward the mould cavities.

**Figure 11:**
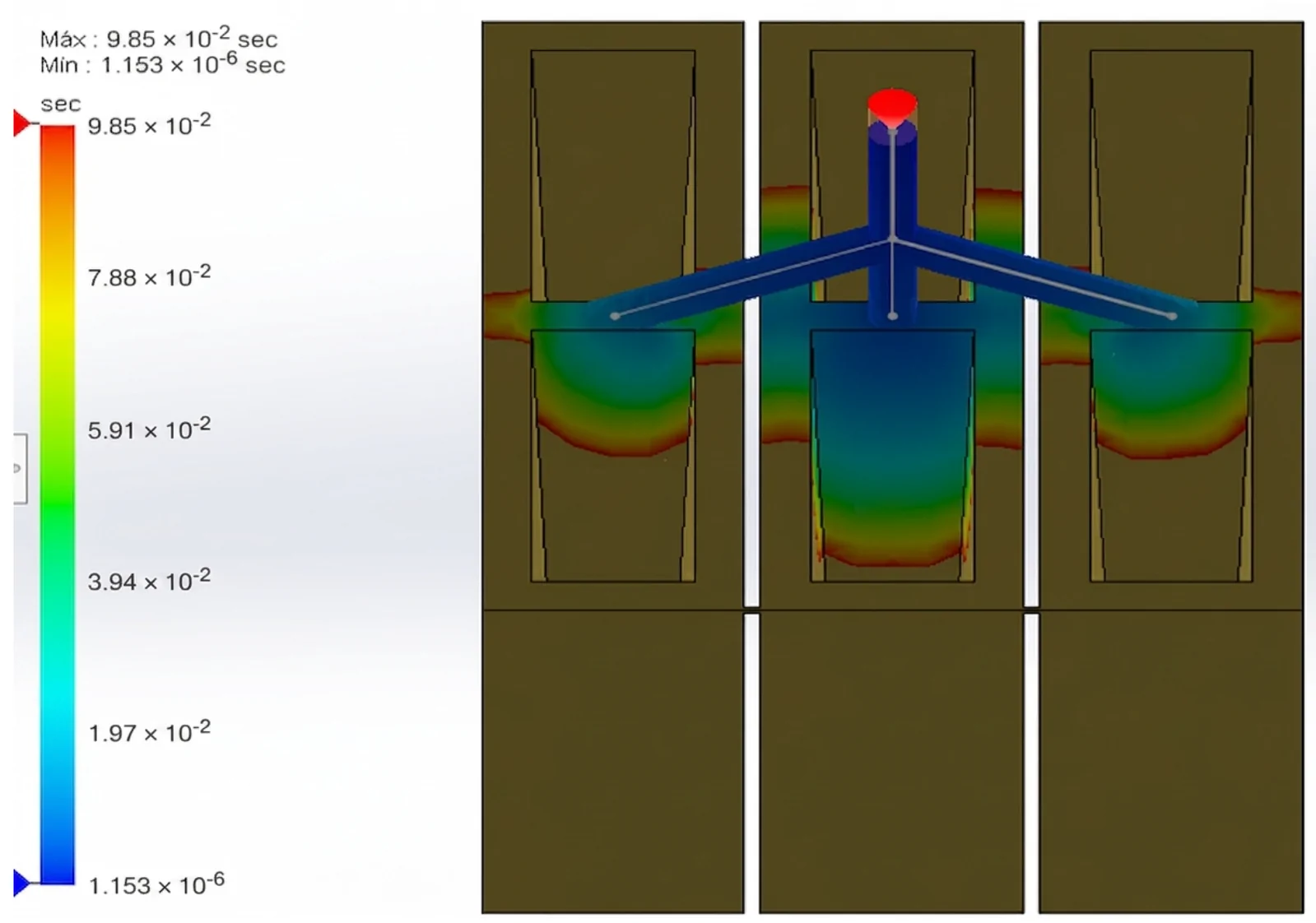
Computational Fluid Dynamics (CFD) rheological simulation illustrates the progression of the flow front within the 10 cm female mould. The colour gradient reflects the filling time, demonstrating a balanced distribution of the viscoelastic concrete mixture.

**Figure 12:**
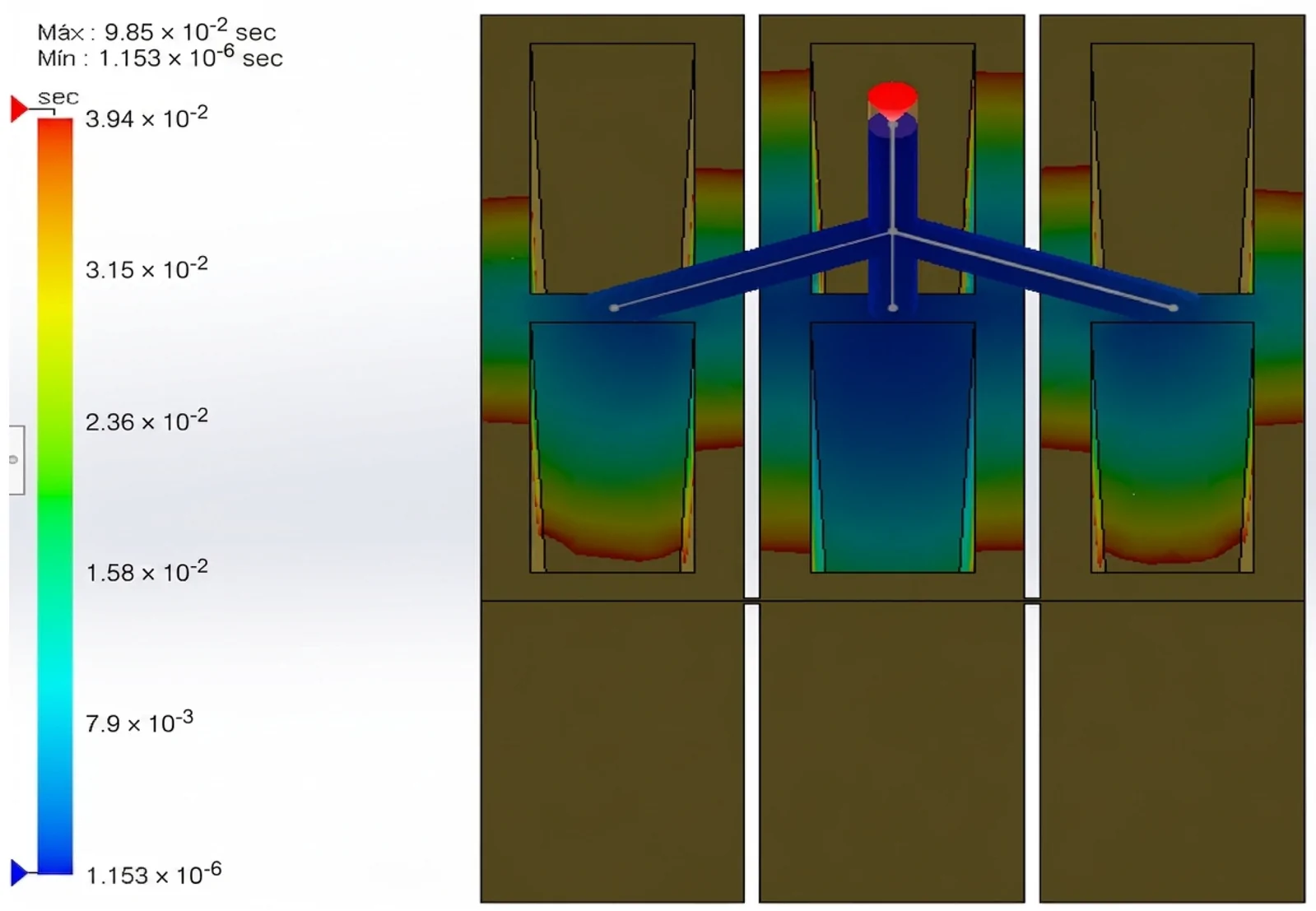
The initial flow of the concrete mix through the piping system into the 10 cm mould cavities was captured at approximately 0.039 s.

**Figure 13:**
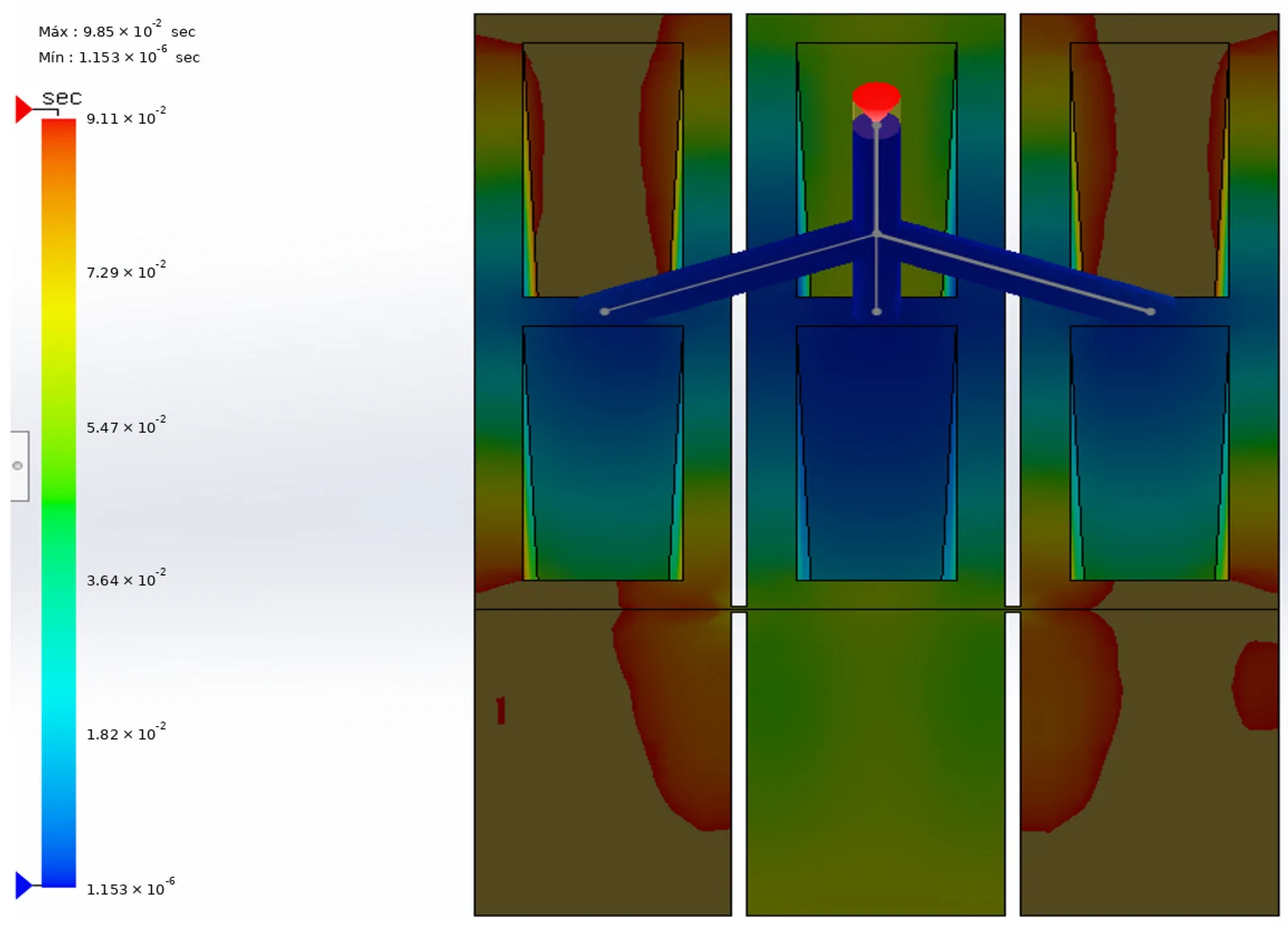
The progression of the final phase of the filling process in the 10 cm female mould was recorded at approximately 0.091 s, indicating that the mixture was on the verge of achieving complete volumetric consolidation.

**Figure 14:**
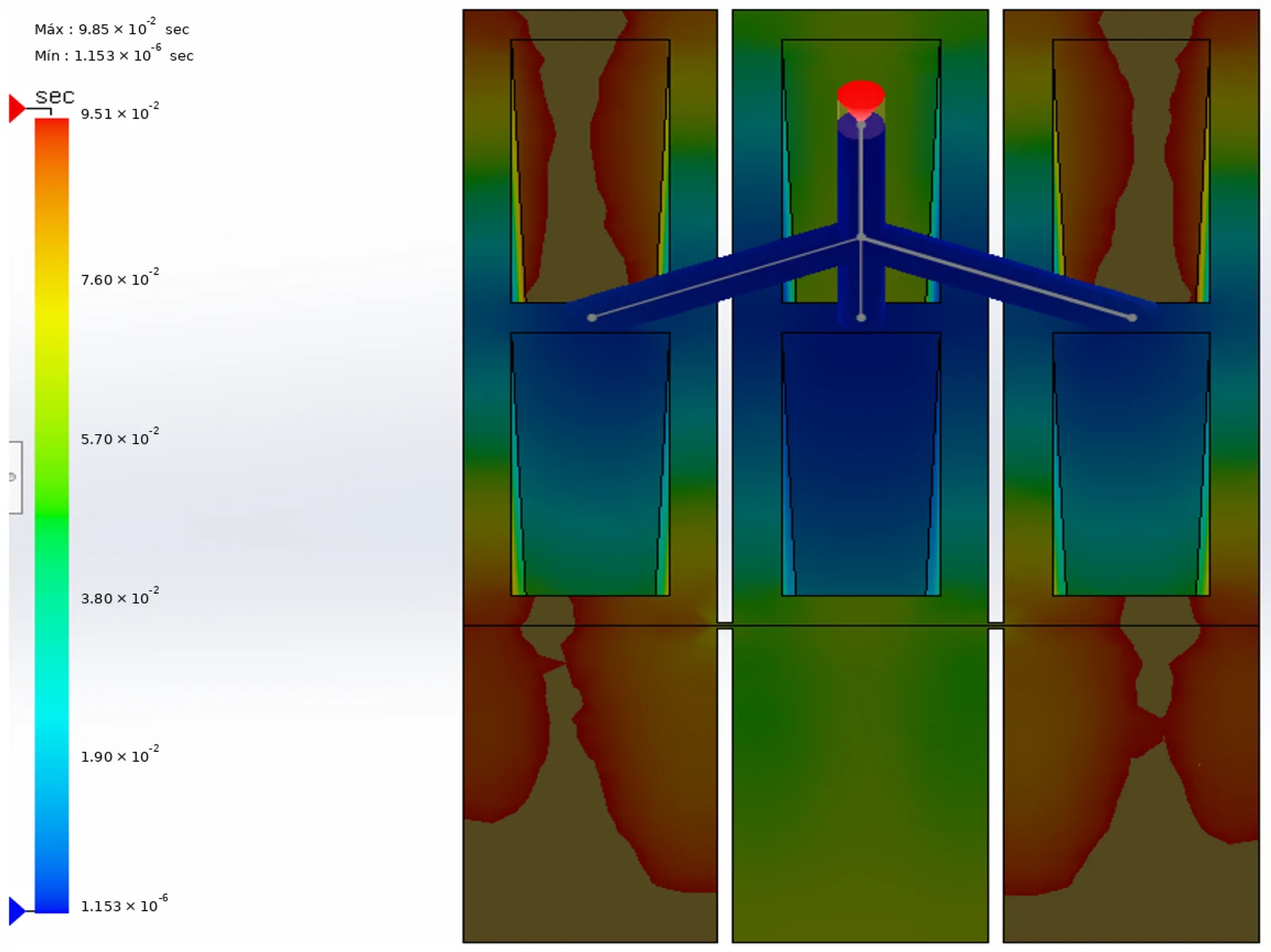
The penultimate phase of the CFD injection cycle was captured at 0.095 s, illustrating the progression of the flow front toward the distal extremities of the 10 cm female die.

**Figure 15:**
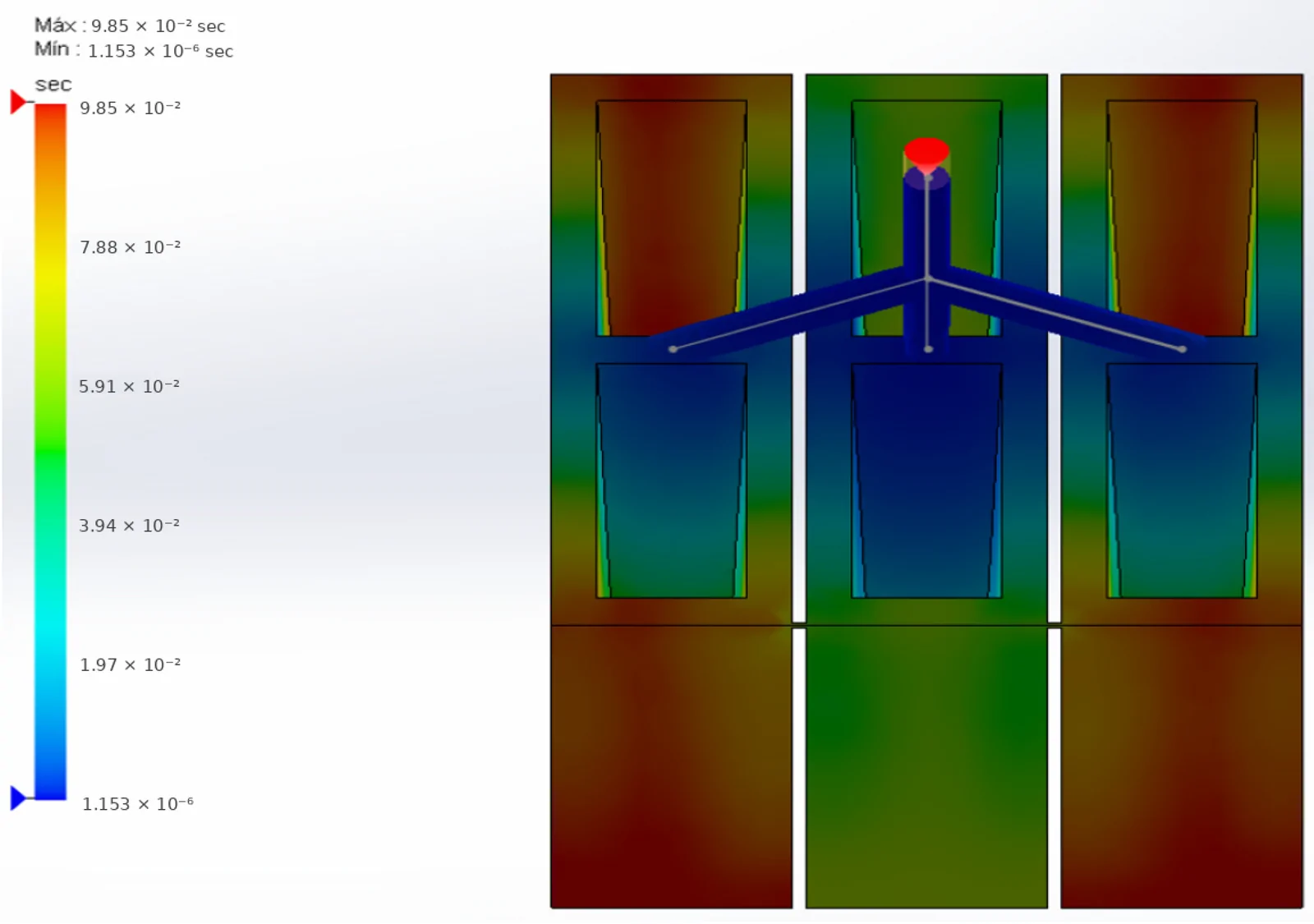
Rheological analysis of the filling time in the 10 cm mould (female die) revealed uniform material distribution within 0.0985 s.

The rheological analysis, executed in SolidWorks Plastics, evaluated the behaviour of the concrete against the parameterised gating system with its 19 mm central injection point (Figure 10, Figure 11, Figure 12, Figure 13, Figure 14 and Figure 15). Two results merit particular attention. First, the maximum filling time remained stable at below 0.11 s regardless of mould size: the mean elapsed time was 0.0985 s for the 10 cm mould, 0.1001 s for the 15 cm mould, and 0.0992 s for the 20 cm mould. Second, in contrast to this time consistency, the energy demand varied significantly with mould volume; the maximum injection pressure required increased from 6.105 MPa for the 10 cm mould, to 10.80 MPa for the 15 cm mould, and 12.90 MPa for the 20 cm mould.

## 4. Discussion

The findings reveal a substantial discrepancy in the distribution of mechanical stress between mould components, which likely accounts for a significant share of the operational failures observed empirically in conventional metalworking practice. The consistently high structural strength of the male punches suggests that their current design is, if anything, oversized. The 10, 15, and 20 cm punches maintain fatigue safety factors greater than 1.0 across the entire evaluated overload range (up to 10,000 N). This indicates a substantial margin under normal operating conditions. This overall robustness leaves room for future weight and material optimisation, an approach with clear precedent in topology-optimised tooling reported for other metal-forming processes, where die and punch weight has been reduced by over 20% without compromising structural performance [25,26,27]. Parametric CAD representations of the kind used here are, moreover, increasingly being coupled with automated and AI-assisted interpretation methods, which may further streamline this design-table approach in future work [28]. In contrast, the vulnerability of the 10 cm female die under cyclic compaction loads exceeding 4000 N indicates that the standard geometries currently employed by the company where this research was conducted frequently enter a fatigue-critical condition. That is to say, the fatigue safety factor computed for the design life of 5 × 10^6^ cycles falls below the required threshold of 1.0. This is indicative of an accumulated effect of repeated loading, rather than immediate static yielding. At these load levels, the von Mises stress remains below the material’s static yield strength (250 MPa). Consequently, the die does not deform plastically under a single application of load. It is hypothesised that the initiation of fatigue cracking will precede the die’s intended service life, consequent to sustained operation at this stress level over multiple compaction cycles.

From a regulatory compliance perspective, these findings, read against the INEN-3066 and ASTM C90 standards, have significant practical implications. Deformation of the mould walls directly alters the dimensions of the finished concrete block, producing units that fall outside tolerance. The proposed parametric model offers two immediate technical responses to this problem. Operationally, as a preliminary measure, it is advisable to calibrate the block-making machine so that the operating force does not exceed 3000 N for the 10 cm format, avoiding permanent deformation. Geometrically, as a second and more durable alternative, the parameterization implemented in SOLIDWORKS makes it possible to modify the thickness of the critical side walls and automatically update the dependent geometry. Each configuration can be evaluated through simulation to determine its effect on the safety factor without the need to redesign the system from scratch [16].

The computational fluid dynamics (CFD) results, in turn, point to a third and complementary approach to manufacturing these components. Confirming that the cavities fill completely in under 0.11 s demonstrates the technical feasibility of transitioning to pressurised concrete-injection systems, which would accelerate production cycles and improve the ergonomics of the process. Future work should prioritise thermal-fatigue analysis of these injection-moulded components, together with experimental validation using physical prototypes subjected to simulated injection pressures of up to 15 MPa.

### 4.1. Proposed Geometric Optimisation

A closer look at the results shows that the 10 cm female die drops below the required fatigue safety threshold at 4000 N, owing to stress concentration within its internal partition walls. Based on the parameterisation already implemented in SolidWorks, an optimised geometric solution is proposed here, consisting of two structural modifications. First, the thickness of the critical side walls (Ep) is increased by 15% for the 10 cm format, moving from the standard commercial dimension to a reinforced thickness that raises the part’s moment of inertia. Second, larger fillet radii or tangential chamfers are introduced at the inner corners of the cavities, removing the 90° angles that currently act as stress concentrators. Because both variables are already defined in the CAD equation tree, this modification triggers an immediate, automated redesign. Based on preliminary simulations of this optimised geometry, it is expected that the critical fatigue load of the 10 cm die (i.e., the load at which the safety factor falls to 1.0) could be raised beyond 4500 N, bringing its fatigue safety factor in line with that of the larger formats and ensuring ample compliance with INEN-3066 without altering the external dimensions of the finished block.

### 4.2. Study Limitations

This study has several limitations that should be acknowledged before its findings are generalised to continuous industrial operation. First, the mechanical properties used for the structural steel, Young’s modulus, Poisson’s ratio, yield strength, and density, were taken from the generic “Structural Steel” material in the ANSYS Engineering Data library rather than from mill-certified test data for the specific tool steel used in production, which may introduce some deviation from the material’s true in-service response. Second, the rheological behaviour of the concrete mixture was modelled as a Bingham plastic material calibrated against standard ASTM C143 slump-test relationships rather than against direct rheometric measurements of the specific mix used by the company, and the simulation results have not yet been validated experimentally against physical prototypes or in-plant trials.

Third, the linear-elastic material model used throughout the structural simulation is only strictly valid up to the yield strength of the steel (250 MPa, Table 4). At the most extreme iterative load evaluated (10,000 N), the von Mises stress in the 10 cm die reaches approximately 390 MPa (Figure 8), exceeding this limit. Beyond the yield point, the actual material response would be governed by plastic deformation rather than the linear-elastic behaviour assumed in the model, so the stress and fatigue-safety values reported for loads above yield should be interpreted as indicative of the trend toward failure rather than as quantitatively precise predictions. The fatigue-safety factors reported within the normal and moderately overloaded range (up to approximately 6175 to 9254 N for the 15 and 20 cm dies, and up to 4000 N for the 10 cm die) remain within the valid elastic regime and are therefore considered reliable.

Fourth, the axial compaction force was represented in the structural model as a uniform pressure distributed over the mould’s flat upper surfaces (Section 2.5), derived from the resultant load predicted by the lever-mechanism analysis. This approach does not address the spatially and temporally varying pressure field that would result from punch movement, direct contact with fresh concrete, high-frequency vibration, and wall friction during actual compaction. The uniform-pressure idealisation is regarded as a satisfactory approach for deriving the global fatigue-critical load levels documented in this study. However, it is acknowledged that this method may result in the under- or over-estimation of localised peak stresses at contact interfaces. It is recommended that a coupled contact-mechanics representation of the punch–concrete–mould interaction, informed by instrumented physical trials, be utilised as a refinement for subsequent, higher-fidelity iterations of this model.

Fifth, a dedicated mesh-convergence (h-refinement) study was not performed at the internal-corner locations where peak stresses were recorded; while the global mesh-quality metrics reported in Section 2.4 (skewness and orthogonal quality) indicate a well-formed mesh overall, localised stress values at idealised sharp corners and abrupt geometric transitions can, in principle, remain mesh-sensitive. The enlarged fillet radii proposed as part of the geometric optimisation in Section 4.1 are expected to reduce this sensitivity in addition to lowering the physical stress concentration, and a formal convergence study at these locations is recommended before the reinforced geometry is finalised for production. Addressing these limitations, through certified material characterisation and physical prototype validation, constitutes a clear priority for future work.

## 5. Conclusions

The development and computational assessment of a parametric CAD-CAE-CFD workflow for concrete block moulds support the following conclusions:

- The utilisation of quality-oriented tools (VOC and QFD) has been demonstrated to be an effective method for translating the dimensional and durability requirements of the INEN-3066 and ASTM C90 standards into controllable CAD variables. The control of wall and partition thickness was identified as the primary design mechanism for ensuring the finished block adheres to the ±3 mm tolerance, thereby supporting the efficacy of a design-table-driven parametric approach over the non-parametric methods currently employed by SMEs in this sector.
- The finite element analysis revealed a marked structural asymmetry between the two halves of the assembly. The male (punch) components demonstrated consistent over-engineering, with fatigue safety factors exceeding 1.61 even under the most extreme overload scenario evaluated (10,000 N). Consequently, they are a natural candidate for future weight and material optimisation. In contrast, the female (die) components represent the critical link: the fatigue safety factor of the 10 cm die falls below the required threshold of 1.0 (reaching a minimum of 0.91) once the compaction load exceeds 4000 N, well within the range of plausible operating overloads. By contrast, the 15 and 20 cm dies tolerate substantially higher loads (6175.6 N and 9254 N, respectively) before the same threshold is crossed.
- To address this vulnerability, a corrective geometric solution is proposed for the 10 cm die, consisting of a 15% increase in critical wall thickness together with enlarged fillet radii at the internal cavity corners. It is evident that the presence of both variables within the CAD equation tree renders this modification as an appropriate candidate for implementation as an automated redesign. Preliminary simulations have been conducted to assess the impact of this modification, and the results indicate that it would result in an increase in the critical fatigue load of the die beyond 4500 N. This outcome would align the performance of the die with that of the larger formats without necessitating any alteration to the external dimensions of the finished block. As a provisional, cost-effective interim measure, it is advised that the compaction force exerted on the 10 cm mould be restricted to below 3000 newtons until the reinforced geometry is implemented
- The rheological simulation confirmed the feasibility of a pressurised concrete-injection process, with the balanced 19 mm gating system filling the mould cavity in under 0.11 s across all three commercial formats. The injection pressure required for this process scaled with mould volume, ranging from 6.105 MPa (10 cm) to 12.9 MPa (20 cm). Beyond its technical feasibility, this ultrafast injection cycle offers a practical route to shortening production cycle times and reducing operator exposure to the vibration and manual handling associated with conventional compaction. This has direct implications for both productivity and workplace ergonomics.
- These conclusions should be considered reflecting the study’s limitations: the structural analysis relies on generic library-grade steel properties rather than mill-certified data, and the concrete rheology was calibrated against standard slump-test relationships rather than direct rheometric measurement of the company’s specific mix. Consequently, experimental validation against certified material data and physical prototypes is imperative before these findings can be generalised to continuous industrial production.

## Figures and Tables

**Table 1:** Summary of the analysed literature on CAD-CAE-CFD integration and the identified research gap.

Reference	Main Findings	Limitations	Research Gap
[8]	CAD/CAE supports parametric modelling and geometric evaluation, reducing the need for physical prototypes.	The approach is general and is not linked to specific regulatory requirements for concrete block moulds.	A standardized parametric workflow aligned with NTE INEN 3066 has not been established.
[9]	Validated finite element analysis estimated a 24.55% reduction in deflection and an 11.57% improvement in load distribution in prestressed concrete bridges.	The study addresses a large-scale civil structure and does not examine industrial tooling at the component level.	The fatigue behaviour of mould components under specific compaction loads has not been assessed.
[10]	Parametric finite element analysis achieved mass reductions of 96.9–99.1% without exceeding the defined stress limits.	The method was applied to generic mechanical components and was not governed by manufacturing standards for concrete blocks.	Existing parametric models do not automatically adapt mould geometry to the structural and dimensional limits established by block standards.
[11]	CAD-integrated finite element analysis evaluated modular connections and reported deflection increases from 0.833 mm to 5.126 mm.	The study focuses on the structural behavior of modular connections rather than on mould design for industrial manufacturing.	An integrated simulation loop that uses structural results to support automated geometric optimization of concrete block moulds is still lacking.
[12]	Finite element analysis of conventional and rubberized concrete blocks showed 45–55% agreement with experimental results.	Geometric simplifications and the assumption of homogeneous material properties contributed to deviations from the experimental results.	Structural simulations need to incorporate accurate cavity geometry and material properties associated with the moulding process.
[13]	CFD models based on Bingham rheology predicted mortar-flow variations associated with slip conditions and contact surfaces.	The rheological analysis was conducted separately from the structural constraints of the mould.	An integrated CAD–CAE–CFD workflow for mould structural and material-flow analysis has not been established.

**Table 2:** CAD design parameters and variables defined in the Equations module.

Symbol	CAD Variable (mm)
L	Total Length
H	Nominal Width
Ep	Wall Thickness
Et	Partition Thickness
Em	Mould Thickness

**Table 3:** Loads applied in the finite element analysis.

Mould Size (cm)	Base Operating Load (N)	Maximum Overload Evaluated (N)
10	1960.0	10,000.0
15	2100.5	10,000.0
20	2254.0	10,000.0

**Table 4:** Mechanical properties of the structural steel used in the finite element analysis (ANSYS default “Structural Steel”).

Property	Symbol	Value
Young’s modulus	E	200 GPa
Poisson’s ratio	ν	0.30
Yield strength	σ_y_	250 MPa
Tensile Ultimate Strength	σ_u_	460 MPa
Density	ρ	7850 kg/m^3^

**Table 5:** Summary of the limit mechanical behaviour in the female dies evaluated in ANSYS.

Mould Size (Female)	Critical Fatigue Load (N)	Max. Deformation (mm)	Von Mises Stress (MPa)	Limit Fatigue Safety Factor ^1^
10 cm Configuration	4000.0	0.20	158.58	0.91
15 cm Configuration	6175.6	0.27	164.25	0.88
20 cm Configuration	9254.0	0.38	157.58	0.92

^1^ Values approximate but do not equal 1.00, corresponding to the nearest simulated load increment at which the threshold is crossed rather than an exact interpolation. The lower value for the 15 cm die reflects local stress-concentration differences, not a non-monotonic trend.

## Data Availability

The original contributions presented in this study are included in the article. Further inquiries can be directed to the corresponding author.

## Abbreviations

CAD	Computer-Aided Design
CAM	Computer-Aided Manufacturing
CAE	Computer-Aided Engineering
FEA	Finite Element Analysis
FEM	Finite Element Method
CFD	Computational Fluid Dynamics
VOC	Voice of the Customer
QFD	Quality Function Deployment
CTQ	Critical to Quality
ASTM	American Society for Testing and Materials
INEN	Instituto Ecuatoriano de Normalización
